# Species-Specific Real-Time PCR Assay for Rapid Identification of *Zeugodacus cucurbitae* Coquillet (Diptera: Tephritidae) from Other Closely Related Fruit Fly Species

**DOI:** 10.3390/insects16080818

**Published:** 2025-08-07

**Authors:** Rebijith Kayattukandy Balan, Sherly George, Gur Pines, Dongmei Li, Disna Gunawardana, Sathish Puthigae

**Affiliations:** 1 Plant Health and Environment Laboratory, Ministry for Primary Industries, 231 Morrin Road, St. Johns, Auckland 1072, New Zealand; sherly.george@mpi.govt.nz (S.G.); dongmei.li@mpi.govt.nz (D.L.); disna.gunawardana@mpi.govt.nz (D.G.); sathish.puthigae@mpi.govt.nz (S.P.); 2 Institute of Plant Protection, Agricultural Research Organization—Volcani Institute, Rishon LeZion 7505101, Israel; gurp@volcani.agri.gov.il

**Keywords:** *Zeugodacus*, invasive, cytochrome oxidase I, real-time PCR, specificity, sensitivity

## Abstract

Fruit flies that belong to the genus *Zeugodacus* (Diptera: Tephritidae) pose significant threats as invasive pests of agricultural crops in Asia and sub-Saharan Africa. The intensification of transboundary trade in fresh horticultural produce has increased the risk of introducing invasive species such as fruit flies, more so through the inadvertent transport of their immature developmental stages. Such immature stages of fruit flies belonging to the Tephritidae family are frequently intercepted at the international borders worldwide and are unable to be identified to the species level using morphological characteristics. Molecular identification using mitochondrial Cytochrome Oxidase I (*COI*) gene has proven to be quite useful, as they are not constrained by developmental stages, sex, or colour morphs of the pest species in question. Also, real-time PCR-based species-specific assays offer quicker turnaround time since they do not require any post-PCR procedures. This study evaluated the utility of a real-time PCR assay based on the *COI* gene region to identify *Zeugodacus cucurbitae* from other Tephritid species. The developed real-time PCR assay provides a swift and precise way of discriminating between these highly invasive pest species during an interception event for rapid decision making. High specificity, having no cross-reactions with closely related Tephritids, and sensitivity of the developed assay will be extremely useful in discriminating *Z. cucurbitae* from other closely related fruit fly species. *Z. cucurbitae*-specific real-time PCR developed in this study is appropriate for organizations that carry out routine diagnostics to facilitate fresh produce imports and exports. Our assay is fully optimized for rapid deployment at international borders, offering reliable detection of the target species regardless of developmental stage, sex, or geographic origins.

## Simple Summary

Fruit flies in the genus *Zeugodacus* are destructive agricultural pests of Asia and expanding to other regions such as sub-Saharan Africa. Growth in international trade and travel increases the inadvertent introduction of pests such as fruit flies, especially through their immature life stages. Immature stages make it challenging to diagnose the organism to the species level using morphology-based taxonomy. In contrast, molecular approaches, particularly COI-based real-time PCR assays, provide swift and precise species identification independent of their sex, life stages, and intactness of the specimen. In the present study, we developed the *Zeugodacus cucurbitae*-specific real-time PCR, with high sensitivity and specificity, to meet international accreditation standards (ISO 17025) and obtained approval to use it through International Accreditation New Zealand (IANZ—ISO 17025).

## 1. Introduction

*Zeugodacus cucurbitae* Coquillet, commonly known as melon fly and native to India is a very serious pest of cucurbit crops [1]. It was first described by Coquillet in 1899 from specimens collected in the Hawaiian Islands and classified it under the genus *Bactrocera*. It is therefore sometimes cited as *Bactrocera* (*Zeugodacus*) *cucurbitae* and is said to be widely distributed in Asia and Africa. It belongs to the Tephritidae family, a diverse collection of true fruit flies that are known for their invasiveness and crop damage [2]. Specifically, fruit flies belonging to the genus *Zeugodacus* (Diptera: Tephritidae) are among the world’s major invasive pests [1] and are highly polyphagous, and this behaviour intensifies their pest status, as it can cause significant economic losses [3].

The female *Z. cucurbitae* lay her eggs beneath the epidermis of host fruits and vegetables, and the larvae develop inside the fruit, leading to tissue damage and making the produce unmarketable [4]. They can infest more than 125 plant species, primarily within the *Cucurbitaceae* family, including various commercially cultivated crops [5,6,7,8,9,10]. Amongst the reported host plants, bitter gourd (*Momordica charantia*), muskmelon (*Cucumis melo*), snap melon (*Cucumis melo* var. *momordica*), and snake gourd (*Trichosanthes anguina* and *T. cucumeria*) are the most preferred [11]. Recently, records from India indicate the expansion of the host range to include tomatoes [12].

A recent investigation into the macrogeographic population dynamics reveals that *Z. cucurbitae* is prevalent across the African continent, the islands of the Indian Ocean, Asia, New Guinea, the Mariana Islands, and Hawaii [10,13]. They successfully invaded Africa in the 20th century, starting in West Africa and progressively spreading eastward, with Mozambique being the most recently documented invasion [7]. *Z. cucurbitae* remain active throughout the year on various hosts, with their spread largely aided by trade, favourable climate conditions, and relaxed biosecurity measures in specific regions.

*Z. cucurbitae* have been encountered at New Zealand’s borders from Asian countries as eggs or larvae and can cause delays in the clearance of fresh produce, as there is currently no rapid and precise test or assay available to identify this pest species. The early detection of exotic insect pest populations, followed by rapid management responses, has resulted in successful control and eradication of numerous species known to inflict ecological or economic damage [14]. A significant challenge is the interception of a large percentage of immature invertebrates (such as eggs, larvae, or pupae) and their body parts that are collected and can be challenging or impossible to identify to the species level using morphological methods. This calls for a method of identification by which a rapid and accurate sex and developmental stage non-limiting inventory of species is achieved, helping classification and management efforts. Such a method would also help in quick and accurate discrimination of native and exotic invasive species at the port of entry which is important from the viewpoint of biosecurity and quarantine.

Molecular diagnostics based on various mitochondrial and nuclear markers is a powerful diagnostic tool [15,16,17,18], as this method is independent of completeness of the specimen, life stages, colour morphs, and sex [19]. DNA-based molecular markers are essential for classifying living organisms, advancing from visible traits to concealed genetic attributes [20]. Mitochondrial markers, particularly the cytochrome c oxidase subunit I (*COI*) gene, have become immensely popular due to their high copy number, rapid evolutionary rate, and lack of introns, rendering them an excellent diagnostic marker among the closely related species [15,21,22,23]. Moreover, the *COI* gene of *Z. cucurbitae* is distinct enough from the other closely related fruit fly species, supporting the design of species-specific assays based on these nucleotide differences. However, PCR-based methods such as PCR-RFLP [24] and barcoding [15] are more time-consuming, as they require post-PCR procedures such as gel electrophoresis and sequencing, respectively. On the other hand, species identification is possible in 2–3 hours’ time with species-specific real-time PCR-based assays, without post-PCR procedures [25]. Such assays have been extensively employed in routine diagnostics around the world for various biosecurity applications, including the identification of invasive insects [26].

In the present study, we developed and tested the *Z. cucurbitae*-specific real-time PCR assay and further validated and accredited it to the ISO 17025 standard by extensive testing to demonstrate the specificity and sensitivity of this developed method. Here we present the development and validation results for both the singleplex and duplex real-time PCR assay (with 18S rRNA as an internal control) for *Z. cucurbitae,* including their sensitivity, specificity, and use of a blind panel test.

## 2. Materials and Methods

### 2.1. Sample Collection and Identification

Specimens of *Z. cucurbitae* at various life stages were obtained from the insect collection resource at the Plant Health and Environment Laboratory (PHEL)—Ministry for Primary Industries (MPI), Auckland, New Zealand, which includes specimens intercepted at our borders and collected from various countries. Additionally, the non-target fruit fly species were also sourced, either as previously extracted genomic DNA or as intact specimens/body parts of the relevant species available in the insect collection at PHEL-MPI. All these specimens were identified by experts at PHEL-MPI either through traditional [27] or integrated taxonomy.

### 2.2. DNA Extraction Method from Fruit Fly Species

Total DNA from each fruit fly specimen was extracted employing the DNeasy Blood and Tissue kit (Qiagen, Valencia, CA, USA). A portion of an adult leg, a piece of larva/pupae, or eggs were utilized for genomic DNA extraction. Specimens were incubated for an hour at 56 °C in 180 µL of ATL buffer and 20 µL proteinase K, and then the extraction was performed according to the manufacturer’s instructions. Amplification competency of all DNA samples was confirmed by real-time PCR amplification of a commercially available 18S rRNA as an internal control (Applied Biosystems; Foster City, MA, USA). A detailed list of species and specimens deployed in our study is provided in Table 1.

### 2.3. PCR Amplification and Sequencing of Fruit Flies

All target species were identified to species either by morphology or by DNA barcoding using LCO 1490 and HCO 2198 primers [28] prior to any further experiments. The PCR reaction contained GoTaq Green master mix (Promega, Madison, WI, USA), 250 nM each of the primers, 0.5 mg/mL BSA, and was conducted using the following PCR cycling parameters: initial denaturation at 94 °C for 5 min; 40 cycles of 94 °C for 15 s, 50 °C for 30 s, 72 °C for 45 s; final extension at 72 °C for 7 min. The amplified products were resolved on 1.0% agarose gel, stained with SYBR™ Safe DNA Gel Stain (Invitrogen, Waltham, MA, USA), and visualized in a gel documentation system (UVP, Ulm, Germany). PCR products were sequenced with the same primers used for PCR. Sanger sequencing of PCR products was performed by EcoGene^®^ (EcoGene, Auckland, New Zealand). All sequences generated from this study have been submitted to NCBI-GenBank under the accession number PV731299–PV731305.

### 2.4. Sequence Analyses

**Figure 1:**
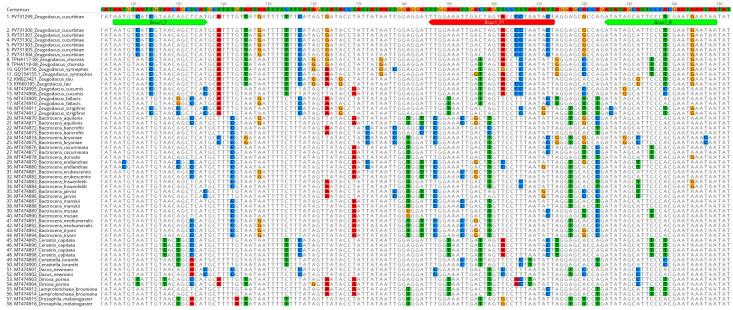
Sequence alignment of *Z. cucurbitae* with phylogenetically related non-target species. The sequences PV731299 to PV731304 represent *Z. cucurbitae* COI sequences obtained from the present study, while the remaining sequences were sourced from the NCBI-GenBank and BOLD databases. Bcuc1_F, Bcuc1_R, and Bcuc1_P1 were the forward primer, reverse primer (both are highlighted in green), and probe (highlighted in red), respectively, developed in this study.

COI sequences generated from this study were assembled (*de novo*) and aligned (MUSCLE alignment) using Geneious Prime 2021.1.1 software [29]. Homology search for *Z. cucurbitae* sequences was carried out using the BLAST programme in NCBI-GenBank (http://www.ncbi.nlm.nih.gov, last accessed on 16 July 2025) and the Barcode of Life Data Systems V4 (BOLD—https://boldsystems.org/, last accessed on 16 July 2025). Furthermore, the sequence variations in the *Z. cucurbitae COI* gene region were determined with other existing sequences in both NCBI-GenBank and BOLD database using the Geneious Prime 2021.1.1 software [29]. COI sequences belonging to various closely related species within Tephritidae were systematically aligned and analyzed to delineate suitable regions for the design of suitable primers and probes (Figure 1).

### 2.5. Z. cucurbitae Real-Time PCR Assay Design

The primer and probe sequences were selected based on the alignment of the partial *COI* gene sequences of *Z. cucurbitae*, and the non-target species, including *Z. chorista*, *Z. synnephes*, *Z. cucumis*, and *Z. tau*. The primers and probes were designed manually, and their secondary structures, along with other thermodynamic characteristics, were analyzed using Geneious Prime 2021.1.1 software and OligoAnalyzer from Integrated DNA Technologies (https://sg.idtdna.com/calc/analyzer, last accessed on 16 July 2025). Such designed primers and probes were further evaluated in Geneious Prime^®^ 2021.1.1 by employing the function for testing specific primers against the chosen sequence or the sequence alignment. We carefully designed primers and probes by ensuring the complete in-silico inclusivity of all known haplotypes of *Z. cucurbitae* so far and maximized in silico exclusivity against the closely related species. The primers and probe employed in this study are provided in Table 2.

### 2.6. Real-Time PCR Assay Optimization

To determine the optimal assay conditions, we conducted our real-time PCR experiments on a CFX96™ Touch real-time platform (BioRad Laboratories Inc., Hercules, CA, USA) with different sets of primers and their corresponding probes at various concentrations (250 nM, 300 nM, and 350 nM). Additionally, the assays were tested with different annealing/extension temperatures (58 °C, 60 °C, and 62 °C) in the presence and absence of BSA (10 mg/mL) and MgCl_2_ (1 mM).

To select the suitable real-time PCR mastermix for our assay, tests were conducted using both PerfeCTa^®^ qPCR ToughMix^®^ (Quanta Bio, Beverly, MA, USA) and SsoAdvanced™ Universal Probes Supermix (BioRad, Laboratories, Hercules, CA, USA). Our new *Z. cucurbitae*-specific real-time PCR was also evaluated in both singleplex and duplex format, using 18S rRNA as an internal control. All assay optimization tests were conducted in two technical replicates.

### 2.7. Sensitivity Assessment

To determine the minimum number of target copies that can be detected by the assay, serial dilutions of an Ultramer^®^ DNA Oligo synthetic template (Integrated DNA Technologies, Singapore) were prepared and tested. Sensitivity or limit of detection (LOD) of the *Z. cucurbitae* COI real-time PCR assay developed was tested with eight serial dilutions (ranging from 10^8^ copies/μL to 10 copies/μL) of the Ultramer^®^ DNA Oligo synthetic templates. All tests for sensitivity were conducted in two technical replicates and the last dilution which resulted in positive amplification in both technical replicates is taken as the LOD for the assay. Furthermore, PCR efficiency, linearity (R^2^), slope, and y-intercept were also determined as part of this assay development.

### 2.8. Specificity Assessment

The specificity of the real-time PCR assay was evaluated by using DNA extracted from various target and non-target species (as shown in Table 1) along with synthetic templates of all those closely related fruit flies whose physical specimens were not available for genomic DNA extraction.

### 2.9. Blind Panel Testing

A total of 34 samples of fruit flies were provided to two scientists for the personalized blind panel testing with no *a priori* information on their origin or identity. Information about the species and specimens subjected to the blind panel test can be found in Table 3. The samples were diagnosed using the duplex real-time assay format, with each sample tested using technical duplicates. The test included both positive and non-template controls during the assay procedure.

## 3. Results

### 3.1. Sequence Analyses and Assay Design

**Figure A1:**
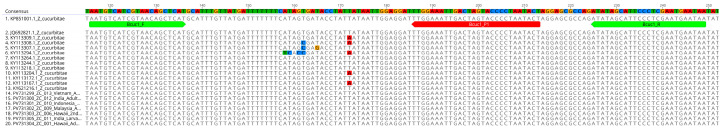
Sequence alignment of various *Z. cucurbitae* haplotypes identified (based on *COI* gene region) within the region of forward and reverse primers (indicated in green). The probe developed in this study is indicated in red. Both the primers and probes matched perfectly to all known haplotypes of *Z. cucurbitae*.

A total of 867 *COI* (5′) gene sequences were downloaded from both NCBI-GenBank and BOLD database (accessed in March 2023), and a total of 13 haplotypes were identified within *Z. cucurbitae* based on Single Nucleotide Polymorphisms (Appendix A, Figure A1). These identified groups were used for further analyses, including primer and probe design. Both the primers and probes were designed manually, after careful consideration of various parameters such as hairpin, self-dimer, and hetero-dimer. The primers and probes thus designed were finally verified using OligoAnalyzer (https://sg.idtdna.com/calc/analyzer, last accessed on 16th July 2025). Primers were designed to yield an amplicon size of 134 base pairs and the designed probe was positioned closer to the reverse primer. While designing the primers and the probes, utmost care was given to avoid the cross-species amplification by including sufficient nucleotide differences in the regions of the primers/probes, while also aiming to amplify all known thirteen haplotypes within *Z. cucurbitae* (Figure 1). Our newly designed primers and probe were also subjected to an evaluation process wherein their in silico specificity was assessed against the sequences of phylogenetically related species (Figure 1) to ensure that they have sufficient mismatches to avoid non-specific amplifications. *Z. cucurbitae* specific primers and probes (Figure 1 and Table 2) thus designed were finally employed in the validation experiments.

**Figure A2:**
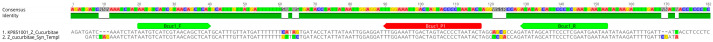
Sequence alignment of *COI* gene region of *Z. cucurbitae* with that of synthetic template positive control of *Z. cucurbitae* indicating the introduced variations that can be used to discriminate the positive control contamination, if required later. Forward and reverse primers are marked in green, while the probe is highlighted in red.

Non-target sequences with less than or equal to four mismatches to the probe sequence were selected for further validation to assess the cross-reactivity. Four *Zeugodacus* species, such as *Z. chorista*, *Z. synnephes*, *Z. cucumis*, and *Z. tau*, which are phylogenetically closer to *Z. cucurbitae,* were selected for further specificity testing. Among these, *Z. chorista* and *Z. synnephes* specimens were not available to us, and consequently, a synthetic template was generated for the COI region encompassing both the forward and reverse primers. Moreover, the plasmids of *Z. cucumis* and *Z. tau*, which carried the COI region as inserts, were utilized in the specificity assessment. The synthetic templates for *Z. cucurbitae* positive control and non-target species were designed based on the target sequence, primers, and probes. Sequence variations were introduced in the synthetic templates so that they can be identified by sequencing at any later date, if required (Appendix A, Figure A2). The synthetic *Z. cucurbitae* positive control was used for assay optimization and was also standardized to use as a positive control in future assays.

### 3.2. Assay Optimization

Both master mixes, namely PerfeCTa^®^ qPCR Toughmix^®^ (QuantaBio, Beverly, MA, USA) and SsoAdvanced™ Universal Probes Supermix (Bio-Rad, Hercules, CA, USA), employed in the initial tests, worked well on the samples tested. *Z. cucurbitae* samples were identified as positive and non-target samples were identified as negative (Appendix B, Table A1 and Table A2). The newly developed *Z. cucurbitae*-specific real-time PCR was further optimized for various primer and probe concentrations (200 nM, 250 nM, and 300 nM) and the ideal concentration of the forward and reverse primers was observed to be 250 nM and that of the probe to be 300 nM. We also tested our assay with and without BSA (10 mg/mL); with and without additional MgCl_2_ (1 mM) as part of optimizing the PCR conditions. Our results indicated that BSA (10 mg/mL) can enhance the assay by preventing non-target amplification, while extra MgCl_2_ had no effect.

Among the various annealing/extension temperatures (58 °C, 60 °C, and 62 °C) tested, 62 °C was found to be the most suited, exhibiting no cross-reactions at all for any of the non-target DNA tested, including *Z. cucumis* plasmid, even at 10^7^ copies/µL concentration. The thermal cycling parameters established for our new assay consist of an initial denaturation phase at 95 °C for 2 min, followed by 40 cycles of the denaturation step at 95 °C for 15 s and an annealing/ extension phase at 62 °C for 45 s, and the assay can be run as a singleplex or duplex format by incorporating 18S rRNA primers and probes from Applied Biosystems™ TaqMan™ Ribosomal RNA Control Reagents (Applied Biosystems, Cat # 4308329, Foster City, CA, USA).

### 3.3. Assay Specificity

The specificity of the newly developed *Z. cucurbitae*-specific real-time PCR was investigated against a range of genomic DNA (newly and previously extracted), plasmid DNA containing the target region, and synthetic construct for target and non-target Tephritids. Most of the specimens employed in the specificity testing originated from different geographical locations. For example, target genomic DNA (including a few from various life stages) of *Z. cucurbitae* was prepared from specimens collected from the USA, India, Malaysia, and Indonesia. A total of 32 non-target Tephritids (as listed in Table 1) have been tested, in both singleplex (Appendix B, Table A1) and duplex (including non-target species tested in blind panel test, Table 3) assay format.

All *Z. cucurbitae* tested produced positive results (with a Cq ≤ 36) and non-target samples produced negative results with no cross-reaction (Table 3). The duplex real-time PCR clearly showed that both target and non-target genomic DNA employed in the specificity assay are PCR-competent (with a Cq value ≤ 30) for 18S rRNA as an internal control (Table 3). Therefore, the newly developed *Z. cucurbitae*-specific real-time PCR is precise enough to identify *Z. cucurbitae* specimens in any given life stage.

### 3.4. Assay Sensitivity

A total of a 169 bp (Appendix A, Figure A2) fragment of the *COI* gene from *Z. cucurbitae* was chemically synthesized and resuspended in Tris EDTA (TE) buffer (Invitrogen, Carlsbad, CA, USA) according to the manufacturer’s instructions. This resuspended synthetic control was used to calculate the copy number and was diluted to 10^8^ copies/µL with TE buffer. From this, ten-fold dilutions were prepared, yielding a final concentration of ten copies, which were used in the *Z. cucurbitae*-specific real-time PCR testing.

**Figure 2:**
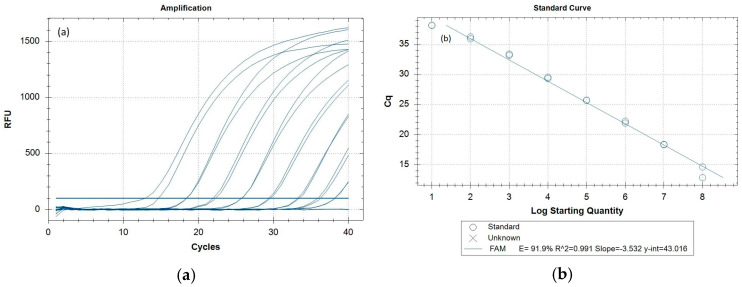
(**a**) Amplification curves for the ten-fold dilution series of the *Z. cucurbitae* synthetic control (10^8^ to 10 copies/µL), employing the PerfeCTa^®^ qPCR Toughmix^®^ (QuantaBio). The baseline threshold was set at 100. (**b**) Standard curve indicating the efficiency of the *Z. cucurbitae*-specific real-time PCR. The standard curve was drawn from the Cq values against the log copy number of 10^8^ to 10 copies/µL of the synthetic control fragments.

The amplification curves, standard curve (Figure 2a,b), and Cq values (Appendix B, Table A3) for the newly developed *Z. cucurbitae*-specific real-time PCR sensitivity test indicate amplification signals observed for the synthetic templates down to 10 copies (though it was amplified at later cycles) (Appendix B, Table A3). The 95% confidence limits of the linear dynamic range are plotted in Figure 2b, with a correlation coefficient, R^2^ of 0.991. The calibration curves also indicated that *Z. cucurbitae*-specific real-time PCR was able to detect the 10 copies/µL concentration of the synthetic template. Insect samples with Cq ≤ 36 are considered positive for *Z. cucurbitae*. Samples with Cq values between 36 and 40 should be further investigated for their accurate species identity. Mostly, while performing the real-time PCR, the Cq values for the positive samples are ≤30 (Table 3). Considering the above fact, we have decided to select 1000 copies of COI target/µL concentration of the synthetic template to provide 100% confidence with the limit of detection (LOD).

### 3.5. Blind Panel Evaluation

A total of 34 insect samples were subjected to blind panel evaluation by two scientists trained in molecular techniques at PHEL-MPI, Auckland. The blind panel tests successfully amplified the target species (*Z. cucurbitae*), as shown in Table 3, while no amplification was observed in any of the non-target species tested. All tested target and non-target genomic DNAs were PCR-competent as demonstrated with a Cq of less than 30 cycles for the *18S rRNA* as an internal control gene.

## 4. Discussion

Fruit flies that belong to the order Tephritidae are recognized worldwide as major invasive pests, posing a significant threat to agricultural production across diverse climates and regions. Increased transboundary movement of agricultural produce has resulted in the chance introduction of many invasive species that include *Zeugodacus* and *Bactrocera* mainly in their immature stages [30,31,32]. Our records at PHEL-MPI show that Tephritid samples are regularly intercepted at the New Zealand border every year as various immature life stages in shipments of fruits and vegetables, which prove to be difficult to identify to the species or even genus level morphologically. Despite numerous identification techniques available for the discrimination of the intercepted pests at immature life stages, morphology-based taxonomic tools are often unreliable and time-consuming, causing significant delays in decision making at the border [24,33]. This demands a quick and accurate species diagnosis method at the port of entry, where the classical taxonomic method has a limited role, as it requires the presence of adult specimens. In this scenario, the molecular methods will be extremely useful as this method is independent of sex, life stages, colour morphs, etc. Considering the economic importance of the fruit fly, several approaches have been tried and tested in the recent past, such as conventional PCR, species-specific PCR, and PCR-RFLP, which have been effective in many ways [19,34,35]. However, such methods are less appropriate for high-throughput or real-time diagnostics due to their labour-intensive nature and the necessity for post-PCR processing. On the other hand, molecular diagnostics based on real-time PCR platforms have transformed the species identification process due to their fast turnaround times, minimal risk of contamination, and being utilized frequently to detect invasive alien insect pests globally [36,37,38].

COI-based DNA barcoding is regarded as one of the most reliable approaches in species discrimination [39] of fruit flies and has proven to be highly effective across various insect orders, including Diptera: Tephritidae [40,41]. Furthermore, it is not merely an identification tool but also enables researchers to verify morphologically similar species and aids in uncovering cryptic species that may be overlooked by traditional methods [18,22,42]. Despite the concerns of its limitations in identifying novel species, issues related to hybridization and regional genetic variation [43,44], the *COI* gene has become the standardized approach in molecular taxonomy since its inception [45]. Recent research has proven effective in employing the *COI* gene for the identification of fruit fly species (Diptera: Tephritidae) [40,41,46], and we also endeavoured to apply the same gene for creating species-specific primers and probes for our real-time PCR assay.

The species-specific real-time PCR assays provide a rapid and accurate diagnosis [25], as such assays are equipped with the MIQE guideline for qualitative assays [47]. Several such real-time PCR assays have been developed so far on various fruit fly species including *Bactrocera* and *Zeugodacus* species [25,48]. Nevertheless, there are currently no real-time PCR assays available for *Z. cucurbitae*, an economically important invasive insect pest that poses challenges in identification during its immature life stages.

The presence of common COI haplotypes across different species of fruit flies introduces additional challenges to ultimate species discrimination [49,50,51]. In the present study, primers and probes were meticulously designed for an amplicon size of 134 base pairs in the *COI* gene region from *Z. cucurbitae* haplotypes, ensuring the complete absence of any non-specific amplification even with the closely related Tephritid species such as *Z. cucumis*, by maximizing in silico exclusivity during primer and probe design [52]. Thus designed, the specificity of our primers and probes ensured that only *Z. cucurbitae* DNA is amplified, avoiding the cross-amplification of DNA from other closely related fruit fly species tested [53].

SYBR green chemistry is known for its simplicity and cost-effectiveness [54,55]. However, the propensity for non-specific amplification remains a significant drawback. Probe-based assays such as those in the present study are known to overcome this issue effectively, and various assays have been successfully developed for many other fruit fly species, such as *Z. cucumis*, *B. jarvisi*, *B. zonata*, and *B. dorsalis* [25,26,56], even though it pushes the cost a bit higher. The *Z. cucurbitae*-specific real-time PCR described here provides a swift and precise way of discrimination of these nasty pest species during an interception event, enabling rapid biosecurity decision making. High specificity (no cross-reactions with closely related Tephritids) and sensitivity of the developed assay will be extremely useful in discriminating *Z. cucurbitae* from the other Tephritids, like how other assays have been designed to differentiate other fruit flies [25,48].

The sensitivity of an assay can be defined as its capacity to identify target DNA at low concentrations, which is of paramount importance for early and reliable identification of invasive pests such as fruit flies. Thus, the high sensitivity reported from our *Z. cucurbitae*-specific real-time PCR facilitates the identification of minimal pest populations prior to the occurrence of outbreaks, thereby enabling timely intervention in its management and eradication [56].

The *Z. cucurbitae*-specific real-time PCR developed in this study is appropriate for any organization carrying out routine diagnostics and facilitating trade. This assay will allow for rapid and accurate discrimination of *Z. cucurbitae* and is fully optimized for immediate deployment at the port of entry in any part of the world, as it can detect the target species from any geographical origin irrespective of its life stages [26,57]. Additionally, the *Z. cucurbitae*-specific real-time PCR developed in this study at PHEL-MPI, New Zealand, received international accreditation and authorization for application through International Accreditation New Zealand (IANZ—ISO 17025) [58].

## 5. Conclusions

Effective biosecurity decisions heavily rely on accurate and efficient diagnostic tests that can identify the species in question, particularly with invasive species like fruit flies, irrespective of their life stages, sex, intactness of the specimen, and colour morphs. The sensitivity, specificity, and efficiency of our newly developed assay ensure precise and rapid species identification of *Z. cucurbitae* from any other Tephritid fruit flies, directly influencing trade, various surveillance programmes, and facilitating effective pest management measures during a response programme. Our assay has shown remarkable suitability for their intended purposes, thereby making them readily adoptable by any other diagnostic laboratories looking to streamline and optimize their *Z. cucurbitae* diagnostics or screening processes for greater efficiency and accuracy in outcomes. In this domain, we have not only developed a *Z. cucurbitae*-specific real-time PCR assay but have also attained international accreditation and approval for its utilization through IANZ—ISO 17025.

## Figures and Tables

**Table 1:** List of specimens employed in the present study.

Sl No	Species ID	Accession No.	Description
	Target species		
1	*Zeugodacus cucurbitae*	BC_001	USA, adult, lab colony
2	*Zeugodacus cucurbitae*	BC_002	Unknown, adult
3	*Zeugodacus cucurbitae*	BC_003	Unknown, pupae
4	*Zeugodacus cucurbitae*	BC_004	USA, adult, lab colony
5	*Zeugodacus cucurbitae*	BC_005	Unknown, 3rd instar larvae
6	*Zeugodacus cucurbitae*	BC_006	USA, 2nd instar larvae
7	*Zeugodacus cucurbitae*	BC_007	USA, adult, lab colony
8	*Zeugodacus cucurbitae*	BC_008	USA, adult, lab colony
9	*Zeugodacus cucurbitae*	BC_009	Malaysia, adult, long bean
10	*Zeugodacus cucurbitae*	BC_010	Indonesia, adult, cue lure trap
11	*Zeugodacus cucurbitae*	BC_011	India, larvae, *Canna edulis*
12	*Zeugodacus cucurbitae*	BC_012	USA, adult, lab colony
13	*Zeugodacus cucurbitae*	T17_02945	India, adult, chilli
14	*Zeugodacus cucurbitae*	T15_7487B	Vietnam, adult
	Non-target species		
1	*Anastrepha* sp.	Ent_38, E6	Peru, mango, larvae
2	*Bactrocera barringtoniae*	BS_009	Unknown, larvae
3	*Bactrocera cacuminatus*	BS_003	Australia, adult, bait trap
4	*Bactrocera carambolae*	BS_006	Indonesia, adult, trap
5	*Zeugodacus chorista*	Not applicable	Synthetic template
6	*Bactrocera correcta*	T15_7487C	Vietnam, adult
7	*Zeugodacus cucumis*	BS_010	Australia, adult, zucchini
8	*Zeugodacus cucumis*	Not applicable	Plasmid
9	*Zeugodacus cucumis*	Not applicable	Plasmid
10	*Zeugodacus cucumis*	Not applicable	Plasmid
11	*Bactrocera curvipennis*	T15_6386	New Caledonia, adult
12	*Bactrocera dorsalis*	T18_3481A	Philippines, egg, paw paw
13	*Bactrocera dorsalis*	T18_2947	India, larva, guava
14	*Bactrocera facialis*	T19_00773	Unknown, adult
15	*Bactrocera invadens*	BQ_24	Kenya, adult
16	*Bactrocera invadens*	T18_2685	India, larvae, mango
17	*Bactrocera jarvisi*	Not applicable	Plasmid
18	*Bactrocera jarvisi*	T17_4198	Australia, pupae, mango
19	*Bactrocera latifrons*	T17_711	Thailand, larvae, chilli
20	*Bactrocera melanotus*	BS_012	Cook island, pupae, mango
21	*Bactrocera nigra*	BS_011	Unknown, adult
22	*Zeugodacus strigifinis*	BS_007	Australia, adult, trap
23	*Bactrocera oleae*	BS_002	Greece, adult, lab colony
24	*Bactrocera passiflora*	T19_229	Fiji, Larvae, chilli
25	*Bactrocera psidii*	BS_004	Samoa, adult, *Syzygium* spp.
26	*Bactrocera psidii*	BS_005	Samoa, adult, *Syzygium* spp.
27	*Zeugodacus scutellata*	T18_2201	China, adult, trap
28	*Bactrocera silvicola*	BS_008	Australia, adult, trap
29	*Zeugodacus synnephes*	Not applicable	Synthetic template
30	*Zeugodacus tau*	Not applicable	Plasmid
31	*Zeugodacus tau*	T16_0199	Unknown, adult, trap
32	*Bactrocera tryonii*	T19_05386	Unknown, adult, trap
33	*Bactrocera tryonii*	T19_00487	Unknown, adult, trap
34	*Bactrocera tryonii*	T19_2213	New Zealand, Trap
35	*Bactrocera umbrosa*	BS_001	Malaysia, adult, M.E. trap
36	*Bactrocera xanthodes*	T19_2976	Fiji, larvae, breadfruit
37	*Bactrocera zonata*	T19_00753	Unknown, larvae
38	*Ceratitis capitata*	CQ_4	USA, adult
39	*Ceratitis capitata*	DN11	USA
40	*Drosophila melanogaster*	T19_3841	Chile, sea container
41	*Drosophila* sp.	T19_5393	Mexico, egg, grape
42	*Drosophila* sp.	T19_5388	Mexico, egg, grape
43	*Drosophila subpulcherella*	DD8	Unknown
44	*Drosophila suzukii*	T19_5260	Unknown, larvae, blue berry

**Table 2:** Primer and probe sequences used in this study.

Target Gene	Primer/Probe Name	Sequence (5′–3′)	Product Size	Reference
Primers to amplify mitochondrial genome regions in *Z. cucurbitae*				
*COI*	LCO 1490	GGTCAACAAATCATAAAGATATTGG	710 bp	[28]
	HCO 2198	TAAACTTCAGGGTGACCAAAAAATCA		[28]
*Z. cucurbitae*-specific real-time PCR assays				
*COI*	Bcuc1_F	AATGTCATCGTAACAGCTCAT	134 bp	Current study
	Bcuc1_R	TTATTCATTCGAGGGAATGCTATAT		Current study
	Bcuc1_P1	FAM-AGTATTAGGGGTACTAGTCAATTTCCAA-BHQ1		Current study

**Table 3:** Results for the blind panel test for *Z. cucurbitae*-specific real-time PCR in duplex format. Samples were tested in technical duplicates for each condition.

Sl. No.	Species ID	Accession No.	Country	Mean Cq (±S.D.) (FAM)	Mean Cq (±S.D.) (VIC)	Result
1	*B. psidii*	BS_004	Samoa	0	24.23 (±0.232)	×
2	*B. psidii*	BS_005	Samoa	0	24.34 (±0.033)	×
3	*B. cucurbitae*	T17_02945	India	15.36 (±0.202)	19.74 (±0.203)	✓
4	*B. psidii*	BS_013	Samoa	0	24.32 (±0.004)	×
5	*B. cucurbitae*	T15_7487B	Vietnam	17.02 (±0.064)	21.39 (±0.028)	✓
6	*B. barringtoniae*	BS_009	PNG *	0	24.23 (±0.049)	×
7	*B. barringtoniae*	BS_014	PNG *	0	24.1 (±0.061)	×
8	*B. barringtoniae*	BS_017	PNG *	0	24.38 (±0.017)	×
9	*B. umbrosa*	BS_001	Malaysia	0	20.03 (±0.164)	×
10	*B. cucurbitae*	BC_005	Unknown	16.07 (±1.159)	20.33 (±1.667)	✓
11	*B. nigra*	BS_011	Unknown	0	20.19 (±0.055)	×
12	*B. nigra*	BS_019	Unknown	0	20.17 (±0.210)	×
13	*B. melanotus*	BS_012	Cook island	0	19.97 (±0.179)	×
14	*B. tau*	T16_0199	Unknown	0	20.14 (±0.021)	×
15	*B. zonata*	T19_0753	Unknown	0	20.01 (±0.158)	×
16	*B. oleae*	BS_002	Greece	0	20.13 (±0.106)	×
17	*B. carambolae*	BS_006	Indonesia	0	25.34 (±0.042)	×
18	*B.nr strigifinis*	BS_007	Australia	0	25.27 (±0.021)	×
19	*B. tryonii*	T19_2213	New Zealand	0	25.33 (±0.066)	×
20	*B. cucurbitae*	BC_010	Australia	14.92 (±1.163)	22.23 (±0.137)	✓
21	*C. capitata*	ERIH#1D1	Hawaii	0	25.38 (±0.084)	×
22	*B. invadens*	T18_2685	India	0	25.15 (±0.276)	×
23	*B. cucumis*	BS_010	Australia	0	25.24 (±0.057)	×
24	*B. cucumis*	BS_020	Australia	0	25.26 (±0.057)	×
25	*B. cucurbitae*	A	Unknown	23.75 (±0.090)	29.43 (±0.029)	✓
26	*B. dorsalis*	B	Unknown	0	23.1 (±0.082)	×
27	*B. tryonii*	C	Unknown	0	29.46 (±0.287)	×
28	*B. cucurbitae*	D	Unknown	18.1 (±0.069)	28.99 (±0.187)	✓
29	*B. facialis*	E	Unknown	0	29.37 (±0.096)	×
30	*B. psidii*	F	Unknown	0	29.48 (±0.138)	×
31	*B. cucurbitae*	G	Unknown	15.8 (±0.190)	18.29 (±0.174)	✓
32	*B. cucumis*	H	Unknown	0	19.14 (±0.249)	×
33	*B. passiflorae*	I	Unknown	0	19.93 (±0.023)	×
34	*B. umbrosa*	J	Unknown	0	24.57 (±0.028)	×

Note: For the *Z. cucurbitae* assay, Cq (FAM) values ≤ 36 are positive. For internal control (IC), samples with Cq (VIC) values ≤ 30 are PCR-competent. Samples with Cq (FAM) values between 36 and 40 need to be further investigated (e.g., PCR). ✓: positive; ×: negative. * PNG—Papua New Guinea.

## Data Availability

Data are contained within the article and Appendix A and Appendix B. The original contributions presented in this study are included in the article. All sequences produced from this research have been submitted to NCBI-GenBank (Accession Numbers—PV731299–PV731305). For additional inquiries, please contact the corresponding author.

## Abbreviations

The following abbreviations are used in this manuscript:

BOLD	Barcode of Life Data Systems
BSA	Bovine Serum Albumin
COI	Cytochrome Oxidase I
IANZ	International Accreditation New Zealand
ISO	International Organization for Standardization
MPI	Ministry for Primary Industries
NCBI	National Center for Biotechnology Information
PCR	Polymerase Chain Reaction
PHEL	Plant Health and Environment Laboratory
RFLP	Restriction Fragment Length Polymorphism
18S rRNA	18S ribosomal RNA

## Appendix B

**Table A1:** Cq values for the newly developed *Z. cucurbitae*-specific real-time PCR singleplex assay employing the PerfeCTa^®^ qPCR Toughmix^®^ (QuantaBio). Samples were tested in technical duplicates for each condition.

Sl. No.	Species ID	Accession Number	Country	Mean Cq (±S.D.) (FAM)	PCR Result
1	*Z. cucurbitae*	BC_005	Unknown	16.15 (±0.287)	✓
2	*Z. cucurbitae*	BC_009	Malaysia	16.21 (±0.211)	✓
**Non-target species**					
1	*Z. chorista*	Not applicable	Synthetic 1 × 10^8^ copies/µL	0	×
2	*Z. chorista*	Not applicable	Synthetic 1 × 10^7^ copies/µL	0	×
3	*B. dorsalis*	T18_3481A	Philippines	0	×
4	*Z. synnephes*	Not applicable	Synthetic 1 × 10^8^ copies/µL	0	×
5	*Z. synnephes*	Not applicable	Synthetic 1 × 10^7^ copies/µL	0	×
6	*B. dorsalis*	T18_2947	India	0	×
7	*Z. cucumis*	BS_010	Australia	0	×
8	*B. melanotus*	BS_012	Cook Island	0	×
9	*Z. cucumis*	Not applicable	Plasmid 1 × 10^9^ copies/µL	0	×
10	*Z. cucumis*	Not applicable	Plasmid 1 × 10^8^ copies/µL	0	×
11	*Z. cucumis*	Not applicable	Plasmid 1 × 10^5^ copies/µL	0	×
12	*B. umbrosa*	BS_001	Malaysia	0	×
13	*Z. tau*	Not applicable	Plasmid 1 × 10^7^ copies/µL	0	×
14	*B. oleae*	BS_002	Greece	0	×
15	*B. jarvisi*	Not applicable	Plasmid 1 × 10^5^ copies/µL	0	×
16	*B. cacuminatus*	BS_003	Australia	0	×
17	*B. psidii*	BS_004	Samoa	0	×
18	*B. carambolae*	BS_006	Indonesia	0	×
19	*B. silvicola*	BS_008	Australia	0	×
20	*B. xanthodes*	T19_2976	Fiji	0	×
21	*Z. scutellata*	T18_2201	China	0	×

Note: For the *Z. cucurbitae* assay, Cq (FAM) values ≤ 36 are positive. Samples with Cq (FAM) values between 36 and 40 need to be further investigated (e.g., PCR). ✓: positive; ×: negative.

**Table A2:** Cq values for the newly developed *Z. cucurbitae*-specific real-time PCR duplex assay employing the PerfeCTa^®^ qPCR Toughmix^®^ (QuantaBio). Samples were tested in technical duplicates for each condition.

Sl. No.	Species ID	Accession Number	Country	Mean Cq (±S.D.) (FAM)	Mean Cq (±S.D.) (VIC)	PCR Result
1	*Z. cucurbitae*	BC_009	Malaysia	16.12 (±0.240)	20.42 (±0.269)	✓
2	*Z. cucurbitae*	BC_008	USA	15.02 (±0.520)	21.42 (±0.025)	✓
3	*Z. cucurbitae*	BC_010	Indonesia	14.32 (±0.647)	18.93 (±0.281)	✓
4	*Z. cucurbitae*	BC_006	USA	16.11 (±0.105)	20.59 (±0.227)	✓
**Non-target species**						
1	*B. facialis*	T19_00773	Unknown	0	22.51 (±0.094)	×
2	*B. zonata*	T19_00753	Unknown	0	20.27 (±0.747)	×
3	*B. invadens*	BQ_24	Kenya	0	19.89 (±0.477)	×
4	*B. tryonii*	T19_05386	Unknown	0	23.57 (±0.341)	×
5	*B. tryonii*	T19_00487	Unknown	0	22.39 (±0.482)	×
6	*B. curvipennis*	T15_6386	New Caledonia	0	22.75 (±0.362)	×
7	*C. capitata*	CQ_04	USA	0	28.2 (±0.002)	×
8	*Anastrepha* sp.	Ent_38, E6	Peru	0	28.15 (±0.131)	×
9	*D. melanogaster*	T19_3841	New Zealand	0	28.45 (±0.071)	×
10	*Drosophila* sp.	T19_5393	Mexico	0	21.74 (±0.196)	×
11	*Drosophila* sp.	T19_5388	Mexico	0	21.04 (±0.113)	×
12	*D. suzukii*	T19_5260	Unknown	0	25.06 (±0.095)	×
13	*B. cucumis*	BS_010	Australia	0	22.51 (±0.709)	×
14	*B. passiflora*	T19_229	Fiji	0	24.8 (±0.130)	×
15	*B. jarvisi*	T17_4198	Australia	0	23.21 (±0.178)	×
16	*B. latifrons*	T17_711	Thailand	0	21.12 (±0.019)	×
17	*B. correcta*	T15_7487C	Vietnam	0	28.46 (±0.207)	×
18	*C. capitata*	DN11	USA	0	20.71 (±0.317)	×
19	*D. subpulcherella*	DD8	Unknown	0	22.1 (±0.198)	×

Note: For the *Z. cucurbitae* assay, Cq (FAM) values ≤ 36 are positive. For internal control (IC), samples with Cq (VIC) values ≤ 30 are PCR-competent. Samples with Cq (FAM) values between 36 and 40 need to be further investigated (e.g., PCR). ✓: positive; ×: negative. S.D.—Standard deviation.

**Table A3:** Cq values for the *Z. cucurbitae*-specific real-time PCR with serially diluted synthetic control from 10^8^ copies to 10 copies/µL. Samples were tested in duplicates for each condition.

Sl. No.	Sample Info	Concentrations	Mean Cq (±S.D.) (FAM)	PCR Result
1	Synthetic template of *Z. cucurbitae*	1 × 10^8^ copies/µL	13.76 (±1.252)	Positive
2	Synthetic template of *Z. cucurbitae*	1 × 10^7^ copies/µL	18.37 (±0.028)	Positive
3	Synthetic template of *Z. cucurbitae*	1 × 10^6^ copies/µL	22.10 (±0.221)	Positive
4	Synthetic template of *Z. cucurbitae*	1 × 10^5^ copies/µL	25.73 (±0.014)	Positive
5	Synthetic template of *Z. cucurbitae*	1 × 10^4^ copies/µL	29.45 (±0.147)	Positive
6	Synthetic template of *Z. cucurbitae*	1 × 10^3^ copies/µL	33.29 (±0.131)	Positive
7	Synthetic template of *Z. cucurbitae*	1 × 10^2^ copies/µL	36.09 (±0.225)	FTR *
8	Synthetic template of *Z. cucurbitae*	10 copies/µL	38.17 (±0.000)	FTR *
9	Water	NTC	0	Negative

Note: All serial dilutions are prepared in TE buffer. FTR *—further testing required for absolute confirmation (for, e.g., PCR). S.D.—standard deviation.
